# Sexual and Bystander Harassment Among Part-Time Employees: Effects on Work Attitudes, Stress, and Withdrawal

**DOI:** 10.3390/bs16010017

**Published:** 2025-12-21

**Authors:** Robert T. Hitlan

**Affiliations:** Department of Psychology, University of Northern Iowa, Cedar Falls, IA 50614, USA; rob.hitlan@uni.edu

**Keywords:** sexual harassment, bystander harassment, part-time employees, work attitudes, work behaviors, job stress

## Abstract

This study investigated the impact of sexual and bystander harassment experiences on work attitudes, stress, withdrawal, and psychological well-being of part-time employees. Participants included 314 female employees who worked part-time in their organizations. All participants completed a computer-administered workplace experience survey assessing various aspects of their work environment, including personal and bystander harassment experiences, work-related attitudes (supervisor satisfaction, coworker satisfaction, and general job stress), work behaviors (work and job withdrawal), and psychological well-being. Experiences of sexual harassment and bystander harassment were predicted to be negatively related to satisfaction and psychological health and positively related to stress and withdrawal. Both forms of harassment were expected to contribute additively to the prediction of work outcomes and psychological health. Moderator models were examined to explore the potential interactions between sexual and bystander harassment. Results indicated that both forms of harassment were related to work attitudes, stress, and withdrawal. Sexual harassment was the strongest predictor of work outcomes. Discriminant Function Analysis provided additional support for group-based distinctions. The results are discussed in terms of interpersonal and organizational implications, limitations, and future research directions.

## 1. Introduction

It has been over 60 years since the passage of Title VII of the 1964 Civil Rights Act, which made discrimination against employees based on race, religion, national origin, color, or gender unlawful. Women’s participation in the workforce has increased substantially since this time. In 1960 the labor force participation rate for women was approximately 38% and reached around 60% by 1999. Since this time, the participation rate has remained relatively stable, with current data indicating a participation rate of approximately 57%, seasonally adjusted (U.S. Bureau of Labor Statistics, 2023, 2025). Women’s inclusion in more varied organizational roles, including professional and managerial roles (Pew Research Center, 2025), has fostered attempts to shift interactions between men and women away from traditional gender roles toward more professional interactions (Sbraga & O’Donohue, 2000). Unfortunately, in many instances, women are still perceived as unwelcome, especially in traditionally male-dominated occupations (Fitzgerald & Cortina, 2018; Gruber, 1989a, 1989b, 1998; Martin & Barnard, 2013). Such perceptions can continue to impact women’s career opportunities and increase the likelihood of withdrawal or quitting their jobs due to chronic anxiety and depression (Bernstein et al., 1992; Cassino & Besen-Cassino, 2019).

Over the past several decades, research on sexual and other forms of harassment has advanced our understanding of the antecedents, characteristics, responses, coping strategies, and short- and long-term consequences of such experiences. Although part-time and contingent employees represent an increasing portion of the labor force, they remain underrepresented in harassment research (McDonald, 2011). Some argue that because part-time employees spend less time on the job, they are less likely to suffer some of the negative outcomes associated with harassment compared to full-time employees. In support of such claims, meta-analyses suggest that part-time employment is often characterized by decreased job demands and less involvement in the day-to-day activities within an organization (Thorsteinson, 2003). However, evidence suggests that part-time work may be associated with more organizational mistreatment, including unwanted sexual attention and sexual harassment (Reuter et al., 2020).

Compared to their full-time counterparts, part-time employees often occupy lower-status positions and have less organizational power and job security and fewer resources available for reporting and coping with harassment and other forms of mistreatment (Jonsdottir et al., 2022; McDonald, 2011; Willness et al., 2007). Part-time employees also tend to occupy roles that require higher levels of customer interaction, exposing them to various forms of organizational mistreatment (Cortina et al., 2002; Reuter et al., 2020; Yagil, 2008). Such mistreatment can negatively impact work attitudes, behaviors, and psychological well-being (Morganson, 2022). However, to date, much less research has focused on this population. The current research fills a gap in the existing literature on harassment by examining the experiences of part-time employees with harassment. This also provides a basis for testing the generalizability of current harassment models.

The legal definitions of harassment are outlined below, along with a discussion of research findings related to work attitudes and behaviors commonly associated with harassment. Meta-analytic research and integrative reviews have shown that experiencing harassment and related types of mistreatment are associated with reduced job satisfaction, poorer evaluations of supervisors and coworkers, increased stress, and increased withdrawal behaviors (Hershcovis, 2011; Hershcovis & Reich, 2013; Schilpzand et al., 2016; Willness et al., 2007). In fact, satisfaction, stress, and withdrawal represent early signs of impaired functioning in organizations and are related to psychological well-being (e.g., emotional exhaustion), behavioral disengagement (e.g., performance decrements), and physical health issues (Bowling & Beehr, 2006). These patterns also align with research by Cortina and Areguin (2021) and Lim and Cortina (2005), demonstrating that part-time employees may be especially vulnerable to resource loss (social and organizational support, social ties) that might otherwise buffer the negative effects of harassment because of their lower status and power and more limited workplace integration.

The conservation of resources (COR) framework provides a basis for understanding how harassment contributes to negative work attitudes, behaviors, and psychological health and why part-time employees may be particularly vulnerable to such experiences (Berdahl, 2007; Hobfoll, 1989; Westman et al., 2004). According to Hobfoll (1989), “the model’s basic tenet is that people strive to retain, protect, and build resources and that what is threatening to them is the potential or actual loss of these valued resources.” (p. 516). Stress often emerges when individuals experience actual or perceived threats to valued resources (e.g., social standing, interpersonal trust, emotional stability, and perceived safety in the workplace). According to Hobfell, individuals with fewer resources, such as part-time employees, are less able to prevent “loss spirals.” Such spirals involve increased employee vulnerability, especially among those with limited resources, and a reduced ability to effectively deal with resource threats (e.g., harassment, rejection, bullying, and other forms of mistreatment; Hobfoll, 1989). Part-time employees often have fewer opportunities to fully develop supportive relationships or organizational ties, making them particularly susceptible to the resource-depleting effects of workplace harassment. This is especially likely when multiple forms of mistreatment co-occur. For example, bystander harassment may further threaten resources and contribute to resource loss, above and beyond the strain of direct sexual harassment experiences, resulting in additional decrements to satisfaction and psychological health and increased withdrawal behaviors and stress (Berdahl, 2007; Westman et al., 2004).

### 1.1. Definition and Frequency of Sexual Harassment

Sexual harassment includes unwelcome sexual advances, touching, sexual jokes, remarks about one’s physical appearance, pressure for dates or attempts to establish sexual or romantic relationships, and other sexualized conduct that is used as a basis for employment or advancement decisions or creates a hostile or intimidating work environment (Fitzgerald et al., 1997b; U.S. Equal Employment Opportunity Commission, n.d.-b). In 2015, the EEOC received approximately 6800 sexual harassment charges (U.S. Equal Employment Opportunity Commission, 2022). While both men and women can experience harassment, data from 2018 to 2021 indicate that approximately 62% of all harassment charges and 78% of sexual harassment charges were filed by women (U.S. Equal Employment Opportunity Commission, 2022, n.d.-a). Most sexual harassment cases involve men harassing women (Sojo et al., 2015).

Such incidents may be driven by men attempting to assert their dominance or reinforce their masculine bravado within a work setting, especially when they perceive their masculinity as being threatened (Hitlan et al., 2009). Traditionally, male-dominated workplaces tend to generate more hostile environment claims, and environments with greater power imbalances report more quid pro quo claims (Hunt et al., 2010; Juliano & Schwab, 2001). In such environments, hostile, degrading, or insulting remarks are often directed toward women because they are women, reflecting misogynistic attitudes and behaviors. (Hardies, 2023). Such instances may also reflect men’s desire to assert dominance over women, which occurs more frequently in traditional male-dominated environments (Gruber, 1989b). Women who challenge or defy this dominance hierarchy and do not behave in gender-stereotypical ways are more likely to become targets and may further fuel male behavior focused on protecting their status and identity (Berdahl, 2007). However, speaking up or behaving more assertively can result in women being labeled as troublemakers or complainers and likely to suffer retaliation. Overall, between 40% and 75% of working women report experiencing sexual harassment at some point in their careers, and these stressors often have a significant impact on their job attitudes and behaviors, performance, and health and well-being (Hardies, 2023; Vijayasiri, 2008).

### 1.2. Sexual Harassment, Work Satisfaction, and Withdrawal

As mentioned above, sexual harassment is related to several facets of satisfaction, including job, coworker, and supervisor satisfaction, which are directly related to withdrawal-oriented behaviors (Cortina & Areguin, 2021; Diez-Canseco et al., 2022; Glomb et al., 1997; Hardies, 2023; Schneider et al., 1997; Willness et al., 2007). Harassment tends to erode various other work attitudes through perceived injustice and emotional exhaustion (Glomb et al., 1997; Lim & Cortina, 2005). Satisfaction is also a key factor in understanding employee motivation, performance, and overall well-being (Gutek & Koss, 1993; Meier & Spector, 2015). The most dissatisfied employees are those most likely to engage in withdrawal-oriented behavior (Laband & Lentz, 1998; Mobley, 1977).

Today, many organizations recognize that satisfaction is vital to employee motivation and an organization’s long-term objectives (Long et al., 2016; Varma, 2017; Riyanto & Herlissha, 2020). Less satisfied employees lack motivation, which impacts their performance (Varma, 2017). Research on nurses has found that stressful work conditions are related to reduced work quality, increased absenteeism, and an increased likelihood of organizational withdrawal (Yeh et al., 2020). Two of the most frequently examined withdrawal-oriented behaviors in the sexual harassment literature are work withdrawal (e.g., absenteeism, tardiness, missing meetings) and job withdrawal (e.g., intent to quit or retire; Hanisch & Hulin, 1990, 1991; Parker & Griffin, 2002). Research also indicates that withdrawal and turnover are common predictors of dysfunctional organizations (Merkin, 2008; Merkin & Shah, 2014), and withdrawal-related behaviors can function as coping mechanisms for workplace mistreatment (Sims et al., 2005). Importantly, the negative effects of harassment can outweigh the positive effects of an otherwise supportive workplace (Kath et al., 2009).

### 1.3. Harassment and Health

Numerous studies have indicated that sexual harassment is correlated with poorer psychological well-being and physical health (Dansky & Kilpatrick, 1997; Fitzgerald et al., 1997a, 1988; Miner-Rubino & Cortina, 2004; O’Connell & Korabik, 2000; Piotrkowski, 1998; Richman et al., 1999; Rospenda et al., 2006; Swim et al., 2001). Women who experience sexual harassment are more likely to report psychological symptoms such as depression and anger (Houle et al., 2011; Vijayasiri, 2008). Early experiences of harassment can have long-term negative effects that persist well into the future, including post-traumatic stress disorder (PTSD)-like symptoms (Gutek & Koss, 1993; Houle et al., 2011). Even brief encounters with harassment or experiencing low levels of harassment (e.g., low frequency of experiences) is sufficient to negatively impact employees’ health (Tomaka & Palacios, 2001; Chan et al., 2008; Maran et al., 2022; Schneider et al., 1997). Additionally, when experienced by younger employees, this distress can contribute to decreased academic performance (Fineran, 2003). The possibility of performance decrements can be applied to many part-time employees, who are often younger than their full-time organizational counterparts.

The studies reviewed above highlight the specific outcomes and processes that are most relevant to the goals of the current research. The reviewed findings provide the conceptual basis for the hypotheses examined in this study and converge on the idea that harassment disrupts the psychological resources needed to adequately cope with such stressors and sustain positive work attitudes, behaviors, and performance. All the studies noted above provide evidence that workplace mistreatment, and more specifically harassment, increases strain and undermines well-being through increased resource demands. These findings also reinforce the idea that harassment likely contributes to dissatisfaction, stress, and withdrawal among both full-and part-time employees.

### 1.4. Beyond Singular Harassment Experiences

Research indicates that direct experiences with sexual harassment usually do not occur in isolation but are part of a larger organizational climate and culture conducive to such behaviors. Organizations prone to one type of harassment are also likely to experience other types (Harned et al., 2002). Bystander harassment refers to an indirect form of sexual harassment in which individuals witness or are aware of others’ harassment in the workplace without being the direct target. In other words, bystanders observe or know about the harassment of others but are not directly involved in the incident (Bowes-Sperry & O’Leary-Kelly, 2005). Such experiences also reflect what others have referred to as ambient sexual harassment, which reflects exposure to a more general climate conducive to harassment (Glomb et al., 1997). Bystander experiences are linked to many of the same negative job-related attitudes and behaviors as direct sexual harassment experiences (Bennett et al., 2017; Jacobson & Eaton, 2018, 2019). In addition, Glomb et al. (1997) and Hitlan et al. (2006) demonstrated that exposure to bystander harassment can negatively affect employees’ well-being and job outcomes, even after controlling for direct harassment experiences. Witnessing or knowing about others’ harassment can also create a climate of fear, lower morale, and reinforce norms of silence (Miner-Rubino & Cortina, 2004; Raver & Nishii, 2010). Bystanders may also experience trauma and heightened perceptions of organizational injustice as a result of their bystander experiences (Miner et al., 2012; Raver & Nishii, 2010).

Employees who witness bystander harassment may feel complicit in an organization’s dysfunction and, as a result, have less respect for their supervisor (O’Leary-Kelly et al., 2009). When combined with direct sexual harassment, bystander experiences may worsen outcomes by reinforcing perceptions of a toxic workplace culture (Bowes-Sperry & O’Leary-Kelly, 2005) and be particularly taxing on one’s available coping resources. A pervasive hostile work environment negatively affects the well-being of all employees, not just those who are directly targeted (Merkin, 2008). Bystander experiences intensify the negative consequences of direct harassment experiences. Employees who personally experience sexual harassment and witness (or know about) others being harassed often report increased helplessness and organizational tolerance for similar behaviors (O’Leary-Kelly et al., 2009; Raver & Nishii, 2010).

Bystander experiences may compound direct harassment experiences, leading to “dual victimization,” draining emotional resources, and increasing disengagement (Hershcovis, 2011; Rospenda et al., 2009). For example, Hitlan et al. (2006) found that even after controlling for direct sexual harassment experiences (e.g., frequency, duration, and number of distinct behaviors experienced), bystander harassment accounted for a significant proportion of additional variance in how upset women were by their direct experiences. Raver and Nishii (2010) and Berdahl and Moore (2006) also found that distinct forms of mistreatment additively contribute to lower levels of satisfaction and increased stress. These findings align with COR theory (Hobfoll, 1989), which proposes that the cumulative effects of experiencing multiple stressors reduce coping resources and increase the likelihood of threat appraisals. Such appraisals are likely to elicit more avoidant or withdrawal-oriented cognitive and behavioral responses, such as decreased satisfaction and psychological well-being and increased work and job withdrawal (Hobfoll et al., 2018). Recognizing different forms and types of workplace harassment is critical to understanding how overlapping experiences can intensify negative outcomes.

Both sexual and bystander harassment reflect exposure to the same underlying organizational climate. As workplace stressors, each contributes to the idea that mistreatment is tolerated and that employees’ social standing or safety may be compromised (Glomb et al., 1997). Therefore, these experiences are expected to draw on similar psychological and emotional resources. Consistent with COR, both types of harassment initiate resource loss spirals through emotional dysregulation and reduced perceptions of organizational fairness (Hobfoll et al., 2018). Accordingly, both were hypothesized to relate similarly to core work outcomes.

### 1.5. Part-Time Employees and Harassment Experiences

Compared to research on full-time employees, much less is known about the experiences and responses of part-time employees to workplace harassment. However, employment statistics indicate that part-time employees represent a significant portion of the US workforce. As of 2024, there were approximately 28 million part-time employees in the United States. This represents approximately 17% of the total workforce. Of these, approximately 17.5 million are women (U.S. Bureau of Labor Statistics, 2024a). Moreover, projections suggest that part-time employment is expected to remain a significant component of the U.S. labor market over the next several years (U.S. Bureau of Labor Statistics, 2024b).

Several characteristics associated with part-time employment further highlight this population’s vulnerability to harassment. In terms of demographic characteristics, part-time employees embody certain characteristics that may make them particularly vulnerable to sexual harassment (Jonsdottir et al., 2022). Part-time employees often have lower levels of organizational power than full-time employees and, as such, often occupy a weaker position within the organization. Such power differentials may create an environment conducive to sexual harassment (Hershcovis & Barling, 2010).

Additionally, similar to other groups of employees (e.g., remote employees; Gajendran et al., 2024), part-time employees may be less likely to develop close connections with supervisors or coworkers, less likely to receive various types of organizational training (Adolfsson et al., 2022), and more likely to experience ostracism within their workplace (Howard et al., 2020). As such, they may have reduced access to coping resources (Adolfsson et al., 2022; Ferreira Sequeda et al., 2018; OECD, 2019). Relatedly, research on employee mistreatment indicates that limited organizational support may heighten vulnerability to negative outcomes resulting from such harassment (Kalleberg, 2000; Rospenda et al., 2009). The often temporary nature of such work can both increase exposure to such behavior and reduce the likelihood that it is reported (Fitzgerald et al., 1995b; Rogers & Henson, 1997). Moreover, meta-analytic evidence indicates that sexual harassment is more prevalent in organizational contexts characterized by power asymmetry (Willness et al., 2007). Such conditions are often associated with part-time employment.

Most theories of sexual harassment have focused on full-time employees, with part-time employees receiving only secondary status (McDonald, 2011). As such, we do not know the extent to which existing models generalize to the population of part-time employees. This group represents a sizeable portion of the workforce, and the current research helps elucidate the experiences and consequences of harassment among part-time employees and, in doing so, contributes to theoretical advancement in this area.

## 2. Overview and Hypotheses

Currently, research on multiple harassment experiences provides support for an additive model. In this model, both forms of harassment (direct sexual experiences and indirect bystander experiences) independently contribute to outcomes. From a COR perspective, sexual and bystander harassment are each expected to decrease key social resources (social support and available coping responses). Such resource decrements are expected to manifest as decreased coworker and supervisor satisfaction. Additionally, the increased emotional demands of such experiences are expected to increase stress and worsen psychological well-being.

COR theory provides a framework for the expectations underlying the direction and pattern of relations across all outcome variables. A multiplicative effect also seems likely for part-time employees, given their employment characteristics. For example, increased power differentials and a lower likelihood of access to training and coping resources may overwhelm the available coping resources for part-time employees. This can result in synergistic or interactive effects on the outcome variables. Consistent with COR theory and previous research in this area, the current study expects cumulative or additive effects of sexual and bystander experiences on satisfaction, stress, psychological health, and withdrawal behaviors (Berdahl & Moore, 2006; Hitlan et al., 2006; Raver & Nishii, 2010; Richman et al., 1999). However, moderating or interactive effects have also been found between different forms of workplace mistreatment and outcomes when examining the effects of ethnic harassment (Schneider et al., 2000) and incivility (e.g., abusive supervision, coworker incivility, customer incivility; Shin et al., 2021). The current study explored the emergence of interaction effects between sexual and bystander harassment experiences among part-time employees. Given the rationale above, the current study examined the following predictions:

**H1.**  *Sexual harassment experiences will be negatively related to supervisor and coworker satisfaction and psychological health, and positively related to job stress, work withdrawal, and job withdrawal.*

**H2.**  *Bystander harassment experiences will be negatively related to supervisor and coworker satisfaction and psychological health, and positively related to job stress, work withdrawal, and job withdrawal.*

**H3.**  *Experiences of sexual and bystander harassment will show additive effects, with each contributing independently to the prediction of satisfaction, psychological health, stress, and withdrawal.*

**H4**  **(exploratory).** *Bystander experiences will moderate the effects of sexual harassment such that the relation between sexual harassment and satisfaction, stress, psychological health, and withdrawal will be strongest for employees experiencing high levels of both sexual and bystander experiences.*

## 3. Method

### 3.1. Participants

Data were collected from a mid-sized public university in the Midwestern region of the United States. The participants included 314 female students who were employed part-time in their organizations. All participants received partial course credit for their participation. The average age of participants was 18.79 (*SD* = 1.44). The majority of participants (95.5%) were Caucasian (n = 300), followed by African American (*n* = 7, 2.2%), Hispanic/Latina (*n* = 3, 1%), Asian or Pacific Islander (*n* = 2, 0.6%), and others (0.6%). The largest percentage of respondents indicated that they were single (96.2%). In terms of workplace gender composition, 2.2% of participants reported working with almost all men, 4.1% reported working with more men than women, 47.8% reported working with approximately equal numbers of men and women, 23.2% reported working with more women than men, and 22.3% reported working with almost all women. The majority (61.8%) indicated that their immediate supervisor was female, and 1.6% reported that they were the first of their sex to hold their positions.

Participants were recruited from undergraduate courses, including Introduction to Psychology, via the Psychology Department’s research participation system (SONA). All participants received partial course credit for their participation. Eligibility required current employment (at least part-time) and age ≥ 18 years. No additional exclusion criteria were applied to the study. All respondents who completed the survey were retained unless they provided incomplete data for the primary study variables. All statistical analyses used listwise deletion to ensure that only complete cases were included.

### 3.2. Measures

#### 3.2.1. Sexual Harassment Experiences

Participant experiences were assessed using the Sexual Experiences Questionnaire (Fitzgerald et al., 1988, 1995a). The SEQ includes 19 items that assess three general categories of sexual harassment, aligned with the EEOC’s legal definition. Participants were asked about their experiences with various social-sexual behaviors involving male coworkers or supervisors at work in the past two years. The SEQ measures three distinct forms of sexual harassment: gender harassment (e.g., “repeatedly told sexual stories or jokes that were offensive to you”; α = 0.72), unwanted sexual attention (e.g., “made unwanted attempts to stroke, fondle, or kiss you”; α = 0.77), and sexual coercion (e.g., “treated you badly for refusing to have sex”). All items were rated on a 5-point frequency scale ranging from 0 (never) to 4 (always). For this sample, Cronbach’s alpha reliability coefficient (based on raw scores) for the full SEQ frequency scale was 0.81.

Participants who reported experiencing at least one SEQ item were asked to indicate how upset they were by the experience(s) on a scale ranging from 0 (not at all) to 4 (extremely upset). This approach allowed for an independent assessment of both the frequency and emotional impact of each experience, enabling a more comprehensive evaluation of whether an incident constituted sexual harassment and its emotional severity (cf. Berdahl & Moore, 2006; Cortina et al., 2002; Fitzgerald et al., 1995a). Participants’ overall sexual harassment scores included only experiences perceived to be at least slightly upsetting. Item scores were calculated by multiplying the reported frequency of each experience by the level of distress caused. This produced an overall impact-weighted harassment score for each item ranging from 0 (no experience or not at all upset by the experience) to 16 (experienced a behavior most of the time (4) and extremely upset (4)). Based on this scaling, the total possible harassment score ranged from 0 to 304 (16 maximum score per item × 19 items = 304, the highest possible score). To simplify interpretation, the summed score was divided by the number of items (19) to create a final overall harassment scale ranging from 0 to 16. For example, a participant who reported experiencing a single harassment item once (1) and rated it as slightly upsetting (1) would have a harassment score of 0.05 on the 0–16 scale ({1 × 1}/19). Thus, the overall sexual harassment scale is best referred to as a weighted exposure index that captures both the frequency and the upsetting nature of harassment experiences.

#### 3.2.2. Bystander Experiences/Stress

The Bystander Experiences/Stress Scale was assessed using the 11-item Bystander Experiences Scale (Schneider, 1996). This scale asks participants to indicate how frequently they are aware of sexual harassment occurring to coworkers within their organization (e.g., “watching or hearing about your coworkers becoming the target of sexist or crude sexual comments”). The five-point frequency response scale was identical to the SEQ, with higher scores indicating more frequent bystander experiences. For this sample, Cronbach’s alpha reliability coefficient (based on raw scores) for the full bystander experiences frequency scale was 0.90.

Following the same scoring logic used for the SEQ, participants who endorsed any items on the Bystander Experiences Scale were asked to indicate how stressed they were by each experience, using a scale ranging from 0 (not at all stressful) to 4 (extremely stressful). Scale scores were calculated using the same multiplication method, creating a weighted exposure index, resulting in a final bystander experience score ranging from 0–16.

#### 3.2.3. Work-Related Attitudes

Satisfaction was measured using the 6-item abridged subscales for supervisor and coworker satisfaction and the 8-item general job stress scale from the Job Descriptive Index (JDI Research Group, 2009; Roznowski, 1989; Smith et al., 1969). For supervisor and coworker satisfaction, respondents were presented with a series of adjectives describing their supervisor (e.g., praises good work, tactful, annoying) and coworkers (e.g., boring, slow, responsible). Participants indicated whether each adjective was descriptive of their supervisor and coworkers using a 3-point response scale (“Yes,” “?”, “No”). Scale scores were created by averaging all subscale items, with higher scores indicating greater supervisor and coworker satisfaction. For this sample, Cronbach’s alpha reliability coefficients (based on raw scores) for the supervisor and coworker subscales were 0.71 and 0.72, respectively. The general job stress scale relied on an identical item presentation (e.g., hassled, pressured, many things stressful) and response format. For this sample, Cronbach’s alpha reliability coefficient (based on raw scores) for the general job stress scale was 0.85.

#### 3.2.4. Work-Related Behaviors

Work withdrawal was assessed using a 12-item version of the Work Withdrawal Scale (Hanisch & Hulin, 1991). This scale measures various withdrawal behaviors, such as being absent, failing to attend meetings, being late, and doing poor-quality work. Responses were obtained on an 8-point frequency scale ranging from 0 (never) to 7 (more than once a week). Scale scores were created by averaging across items, with higher scores indicating greater withdrawal from work. For this sample, the Cronbach’s alpha reliability coefficient (based on raw scores) was 0.78. Job withdrawal represents an individual’s intention to leave or quit their job. This was measured using a 3-item modified version of Hanish and Hulin’s job withdrawal scale, which assesses how often respondents thought about quitting their job, the likelihood of quitting, and how desirable it would be. Responses were measured using a 5-point scale ranging from 1 (very unlikely/undesirable) to 5 (very likely/desirable). Scale scores were created by averaging the scores across all items. For this sample, Cronbach’s alpha reliability coefficient (based on raw scores) for the job withdrawal scale was 0.73.

#### 3.2.5. Psychological Health and Well-Being

Psychological health was measured using a modified 13-item version of the Mental Health Inventory (MHI; Veit & Ware, 1983). This scale was designed to assess respondents’ positive affect (e.g., “How much of the time have you felt that the future looks hopeful and promising?”), emotional ties (e.g., “How much of the time have you felt loved and wanted?”) and well-being (e.g., “How much of the time have you generally enjoyed the things you do?”). Responses were obtained on a 6-point frequency scale ranging from 1 (none of the time) to 6 (all of the time). Scale scores were created by reverse coding (as appropriate) and averaging across items. Higher scores indicate better psychological health. For this sample, Cronbach’s alpha reliability coefficient for psychological health was 0.89.

#### 3.2.6. Control Variable

Because all data were obtained from a single source at a single point in time, it was important to reduce the possibility of common method bias (Podsakoff et al., 2003). Additionally, Reknes et al. (2019) found that affective disposition is related to employees’ reactions to workplace environments. Affective disposition was assessed using the 13-item Satisfaction with Neutral Objects Questionnaire (Weitz, 1952). This scale includes items measuring participants’ satisfaction with neutral objects (e.g., the size of a refrigerator and the color of a stop sign). Participants responded on a three-point scale: satisfied, neutral, or dissatisfied. Higher scores indicate a more negative affective disposition. The reliability of this scale has been supported by previous research (Judge & Hulin, 1993; Weitz, 1952). For this sample, Cronbach’s alpha reliability coefficient for affective disposition was 0.81.

### 3.3. Procedure

All participants completed a computer-based workplace experience survey. Before completing the study, all participants were asked to read and sign an informed consent sheet indicating that the survey would ask about various aspects of their workplace environment, including workplace stressors. Participants were assured that their responses would remain confidential. All study procedures were reviewed and approved by the Institutional Review Board of the University (Protocol #L-21-0049). All procedures complied with university and federal ethical guidelines for research involving human participants.

Because harassment experiences typically occur at low base rates, these variables often display substantial positive skew, which can violate the assumptions required for regression-based analyses (Berdahl & Moore, 2006). To address this, distributions were initially checked for violations of assumptions, and data transformations were applied to normalize the distributions where appropriate.

To examine the main effects and interactions, analyses were conducted using the PROCESS macro and SPSS (version 29). The PROCESS (v5.0) macro is a widely used plug-in for estimating the additive and interaction effects among predictors (Hayes, 2022). In addition, discriminant function analysis (DFA) was conducted as an exploratory complement to the regression models. DFA evaluates whether participants can be correctly classified (as a function of work attitudes and behaviors and psychological well-being) based on harassment experiences.

## 4. Results

### 4.1. Preliminary Analyses

This analysis indicated that the distribution of direct sexual harassment was severely skewed (4.12, *SE_skew_* = 0.138, *z* = 29.86, *p* < 0.001) and kurtotic (22.02, *SE_kurtosis_* = 0.275, *z* = 80.07, *p* < 0.001). A similar distribution emerged for bystander sexual harassment experiences (*skew* = 5.41, *SE_skew_* = 0.139, *z* = 38.92, *p* < 0.001; *kurtosis* = 36.14, *SE_kurtosis_* = 0.276, *z* = 130.94, *p* < 0.001). To normalize each of these distributions, a Yeo-Johnson transformation was conducted. The transformation resulted in a substantial reduction in skewness and kurtosis for both direct sexual harassment (*skew* = 1.03, *SE_skew_* = 0.138; *kurtosis* = −0.558, *SE_kurtosis_* = 0.275) and indirect bystander experiences (*skew* = 1.31, *SE_skew_* = 0.139; *kurtosis* = 0.013, *SE_kurtosis_* = 0.276). Additionally, supervisor satisfaction and coworker satisfaction were both significantly negatively skewed: supervisor (*skew* = −1.15, *SE_skew_* = 0.138, *z* = 8.33, *p* < 0.001; *kurtosis* = 0.510, *SE_kurtosis_* = 0.274, *z* = 1.86, *p* > 0.05) and coworker (*skew* = −1.54, *SE_skew_* = 0.138, *z* = 11.16, *p* < 0.001; *kurtosis* = 2.27, *SE_kurtosis_* = 0.274, *z* = 8.28, *p* < 0.001). As a result, these variables were transformed via a reflect and log transformation, resulting in a substantial reduction in non-normality after transformation: supervisor (*skew* = 0.531, *SE_skew_* = 0.138, *z* = 3.85; *kurtosis* = −0.715, *SE_kurtosis_* = 0.274) and coworker (*skew* = 0.824, *SE_skew_* = 0.138, *z* = 5.97; *kurtosis* = −0.293, *SE_kurtosis_* = 0.274). Finally, work withdrawal showed a positive skew (*skew* = 1.14, *SE_skew_* = 0.138, *z* = 8.26, *p* < 0.001; kurtosis = 1.48, *SE_kurtosis_* = 0.274, *z* = 5.40, *p* < 0.001). After log transformation, work withdrawal was normalized (*skew* = 0.451, *SE_skew_* = 0.138, *z* = 3.27; *kurtosis* = −0.124, *SE_kurtosis_* = 0.274, *z* = 0.452). Subsequent analyses were performed on these transformed variables. Given the reflect and log transformation for supervisor and coworker satisfactions, the interpretation of both is the direction opposite their initial scoring. Higher transformed values now reflect lower levels of supervisor and coworker satisfaction.

In addition, given that previous research indicates that sexual harassment is more prevalent in male-dominated environments (Gruber, 1989b; Hardies, 2023), workplace gender composition was examined for its impact on sexual and bystander-harassment experiences. The results of a one-way MANOVA did not indicate a significant multivariate main effect for workplace composition after controlling for affective disposition, Wilks Λ = 0.963, *F*(10, 592) = 1.13, *p* = 0.34, η^2^ = 0.019. Consistent with this multivariate result, univariate ANOVA follow-up tests indicated that neither the main effect for direct sexual harassment, *F*(5, 297) = 1.99, *p* = 0.079, partial η^2^ = 0.033, nor bystander experiences, *F*(5, 297) = 0.94, *p* = 0.45, partial η^2^ = 0.016, were significant. This pattern suggests that workplace gender composition did not contribute to either sexual or bystander harassment experiences in the current sample of part-time employees. This also suggests that any obtained relationship between harassment and outcomes in the current analyses is not due to gendered workplace composition.

### 4.2. Frequency of Harassment

Table 1 lists the frequencies of the different types of direct sexual harassment experienced by the participants. Overall, 39.5% (*n* = 122) reported experiencing at least one direct instance of sexual harassment that was at least slightly upsetting. Among those who reported direct sexual harassment experiences, the largest percentage indicated that their experiences included both gender harassment and unwanted sexual attention, followed by those who reported experiencing only gender harassment. In contrast, the fewest number of participants reported sexual coercion either by itself or in combination with other forms of direct sexual harassment (*n* = 10, 3.1%), and eight participants reported experiencing all three forms of direct sexual harassment (see Table 1).

To further assess participants’ experiences with sexual and bystander harassment, mutually exclusive groupings were created. Table 2 presents the frequency breakdown of these groups. Of those reporting harassment experiences, the largest proportion of employees reported experiencing both direct sexual and bystander harassment. In contrast, smaller proportions reported experiencing only direct sexual harassment or only bystander harassment. Based on these frequencies, participants who reported experiencing one form of harassment were also likely to experience other forms.

### 4.3. Correlations Among Variables

Table 3 presents the zero-order correlations, means, and standard deviations for the study variables. Because several variables were transformed to address non-normality, higher values on the reflect and log-transformed coworker and supervisor satisfaction scales indicate lower satisfaction. Higher values on the transformed work withdrawal and harassment variables are interpreted the same as their raw score values (higher values reflect more work withdrawal and harassment).

Correlational analyses revealed a significant positive correlation between direct sexual harassment and bystander sexual harassment experiences, supporting the frequency-based results and indicating that a climate conducive to one form of harassment is conducive to other forms as well. In partial support of Hypotheses 1 and 2, employees who reported higher levels of sexual harassment also reported lower levels of supervisor satisfaction and higher levels of work-related stress, job withdrawal, and work withdrawal. A similar pattern emerged for bystander harassment experiences. Higher levels of bystander experiences were associated with lower supervisor satisfaction and increased work-related stress, work withdrawal, and job withdrawal. However, neither sexual nor bystander harassment experiences were significantly related to coworker satisfaction or psychological health. The similarity in patterns of relations indicates that sexual and bystander harassment experiences each accounted for similar portions of variability in attitudes and behaviors, although direct sexual harassment had numerically stronger relations across all variables.

### 4.4. Tests of Additive and Interaction Effects

To examine the hypothesis that sexual harassment and bystander experiences would contribute additively (H3) and interactively (H4 exploratory) to the prediction of satisfaction, stress, psychological well-being, and withdrawal, a series of moderated regression analyses were conducted using Hayes (2022) PROCESS macro. The main effects and interaction terms for each analysis were simultaneously entered. In addition, affective disposition was used as a control variable within each analysis to control for the generalized tendency to view various types of interactions negatively. Moreover, given the significant relation between affective disposition and sexual harassment experiences, *r*(302) = 0.13, *p* < 0.05; coworker satisfaction, *r*(302) = 0.13, *p* < 0.05; work stress, *r*(302) = 0.13, *p* < 0.05; and psychological well-being, *r*(302) = −0.23, *p* < 0.01, both coworker satisfaction and psychological well-being were retained and analyzed to determine whether harassment predicted coworker satisfaction after accounting for individual differences in affective style.

#### 4.4.1. Supervisor Satisfaction

For supervisor satisfaction, the overall model was significant, *F*(4, 299) = 6.71, *p* < 0.001, *R*^2^ = 0.08. Sexual harassment predicted supervisor satisfaction, *b* = 0.10, *SE* = 0.03, *t*(299) = 3.55, *p* < 0.01. However, bystander harassment was not related to supervisor satisfaction, *b* = −0.00, *SE* = 0.03, *t*(299) = −0.07, *p* = 0.94. In addition, the interaction term between sexual and bystander harassment was not significant, Δ*R*^2^ = 0.001, *F*(1, 299) = 0.31, *p* = 0.58. Affective disposition was not related to supervisor satisfaction, *b* = 0.07, *SE* = 0.07, *t*(299) = 0.94, *p* = 0.35. Thus, even after controlling for affective disposition, sexual harassment predicted decreased supervisor satisfaction.

#### 4.4.2. Coworker Satisfaction

The overall model predicting coworker satisfaction was not significant, *F*(4, 299) = 1.98, *p* = 0.10, *R*^2^ = 0.03. Neither sexual harassment, *b* = 0.05, *SE* = 0.03, *t*(299) = 1.64, *p* = 0.10 nor bystander harassment, *b* = −0.02, *SE* = 0.03, *t*(299) = −0.67, *p* = 0.50 significantly predicted coworker satisfaction. The interaction between sexual and bystander harassment was also not significant, Δ*R*^2^ = 0.000, *F*(1, 299) = 0.01, *p* = 0.91. However, affective disposition negatively predicted coworker satisfaction, *b* = 0.14, *SE* = 0.07, *t*(299) = 2.05, *p* = 0.04.

#### 4.4.3. Job Stress

The overall job stress model was significant, *F*(4, 299) = 8.21, *p* < 0.001, *R*^2^ = 0.10. Sexual harassment was a significant positive predictor of job stress, *b* = 0.23, *SE* = 0.07, *t*(299) = 3.48, *p* < 0.001, indicating that higher levels of harassment were associated with greater stress. Bystander harassment was unrelated to job stress, *b* = 0.07, *SE* = 0.07, *t*(299) = 0.93, *p* = 0.35. In addition, neither the interaction between sexual and bystander harassment, Δ*R*^2^ = 0.0001, *F*(1, 299) = 0.05, *p* = 0.82, nor was the relation between affective disposition and job stress, *b* = 0.26, *SE* = 0.16, *t*(299) = 1.66, *p* = 0.10, were significant. Thus, after controlling for affective disposition, sexual harassment was found to be the only significant predictor of job stress.

#### 4.4.4. Psychological Health

For psychological health, the overall model was significant, *F*(4, 299) = 5.25, *p* < 0.001, *R*^2^ = 0.07. Neither sexual harassment, *b* = −0.09, *SE* = 0.06, *t*(299) = −1.61, *p* = 0.11, nor bystander harassment, *b* = 0.09, *SE* = 0.06, *t*(299) = 1.42, *p* = 0.16, predicted psychological health after controlling for affective disposition. The interaction between sexual and bystander harassment was also not significant, Δ*R*^2^ = 0.001, *F*(1, 299) = 0.45, *p* = 0.50. However, affective disposition predicted psychological health, *b* = −0.54, *SE* = 0.14, *t*(299) = −3.98, *p* < 0.001. Higher negative affect scores predicted worse psychological health.

#### 4.4.5. Work Withdrawal

For work withdrawal, the overall model was significant, *F*(4, 299) = 4.94, *p* < 0.001, *R*^2^ = 0.06. Sexual harassment was significantly associated with greater work withdrawal, *b* = 0.05, *SE* = 0.02, *t*(299) = 2.85, *p* < 0.01. Bystander harassment was not a significant predictor of work withdrawal, *b* = 0.03, *SE* = 0.02, *t*(299) = 1.29, *p* = 0.20. Additionally, the interaction between sexual and bystander harassment was not significant, Δ*R*^2^ = 0.003, *F*(1, 299) = 1.00, *p* = 0.32. Affective disposition was also not predictive of work withdrawal, *b* = 0.02, *SE* = 0.04, *t*(299) = 0.40, *p* = 0.69. Thus, the only significant predictor of work withdrawal in this model was sexual harassment.

#### 4.4.6. Job Withdrawal

The overall model for job withdrawal was significant, *F*(4, 299) = 4.23, *p* < 0.01, *R*^2^ = 0.05. Sexual harassment significantly predicted higher job withdrawal, *b* = 0.20, *SE* = 0.07, *t*(299) = 2.92, *p* < 0.01, whereas bystander harassment was unrelated to job withdrawal, *b* = 0.03, *SE* = 0.08, *t*(299) = 0.35, *p* = 0.72. The interaction between sexual and bystander harassment was not significant, Δ*R*^2^ = 0.0009, *F*(1, 299) = 0.29, *p* = 0.59. Affective disposition was also not predictive of job withdrawal, *b* = 0.21, *SE* = 0.17, *t*(299) = 1.23, *p* = 0.22.

### 4.5. Supplemental Discriminant Function Analysis

To more fully investigate whether the set of work-related attitudes and behaviors could reliably differentiate between participants depending on their harassment experiences, a discriminant function analysis (DFA) was conducted (Huberty & Olejnik, 2006; Klecka, 1980). Participants were classified into one of four mutually exclusive groups: (1) no harassment, (2) sexual harassment only, (3) bystander harassment only, and (4) those experiencing both forms of harassment. The predictor variables were supervisor satisfaction, coworker satisfaction, job stress, psychological well-being, job withdrawal, and work withdrawal.

A preliminary screening was conducted before concluding the DFA to determine whether the data met the assumptions underlying the DFA. The results indicate that the assumptions for conducting a DFA were met. The intercorrelations among the predictors showed no evidence of multicollinearity (all |*r*| < 0.44). Additionally, *Box’s M* test indicated that the assumption of covariance matrix homogeneity was satisfied, *M* = 74.65, *F*(63, 8028.64) = 1.08, *p* = 0.31. Next, the assumption of multivariate normality was assessed using the within-group Mahalanobis distance (df = 6, *p* < 0.001, critical χ^2^ = 22.46). No cases exceeded the critical value, indicating that there were no multivariate outliers and that the assumption of multivariate normality was not violated. Additionally, all group sizes exceeded the number of predictors, and inspection of scatterplots suggested approximate linear relationships among predictors within groups. Taken together, these results support the use of DFA.

Three discriminant functions were derived (Table 4); however, only the first function was statistically significant. The first function had an eigenvalue of 0.17 and a canonical correlation of 0.38 (*R*^2^ = 0.14), accounting for 80.2% of the between-group variance (Wilks’s Λ = 0.82, χ^2^(18) = 61.14, *p* < 0.001). Table 5 and Table 6 show the standardized canonical discriminant function and structure matrix coefficients, respectively. After controlling for other predictors in the model, the standardized coefficients represent the unique relation between each predictor and each function. The structure coefficients indicate the bivariate relation between each predictor and the discriminant function. This matrix indicated that job stress (0.68), work withdrawal (0.59), supervisor satisfaction (0.55), and job withdrawal (0.54) contributed the most to the between-group separation for the first significant function. In contrast, psychological health (−0.15) and coworker satisfaction (0.05) showed only weak, non-significant loadings. This function was labeled “organizational stress, supervisor satisfaction, and withdrawal.” Additionally, the group centroids indicated that this function maximally distinguished the no harassment (−0.33) and bystander-only groups (−0.40) from the sexual harassment (0.36) and sexual and bystander groups (0.59). The sexual and bystander groups had the highest scores on Function 1, meaning that this group reported the lowest supervisor satisfaction and the highest levels of stress and withdrawal.

As shown in Table 7, the model correctly classified 58.6% of the cases, with a cross-validated accuracy of 57.9% using the leave-one-out method (Lachenbruch, 1967; Lachenbruch & Mickey, 1968). This exceeded the proportional-by-chance rate of 39.4% and the 1.25 × chance criterion of 49.3% (Huberty, 1994; Press & Wilson, 1978). Furthermore, a Press’s Q test indicated that classification accuracy was significantly greater than chance, *Q*(3) = 17.7, *p* < 0.01 (Press & Qureshi, 1973; Tabachnick & Fidell, 2019).

### 4.6. Analyses Summary

Overall, these findings indicate that sexual harassment experiences are most strongly related to adverse work attitudes, stress, and withdrawal. Moderated regression analyses and DFA sexual harassment predicted worse outcomes, even after controlling for employees’ general negative dispositional tendencies. Additionally, the DFA results indicated that the combined sexual and bystander harassment group scored highest on the first function: organizational stress, supervisor satisfaction, and withdrawal, indicating greater stress and withdrawal and lower satisfaction. These findings are consistent with the literature on cumulative stressors and resource depletion (Bowling & Beehr, 2006; Cortina & Magley, 2003; Hobfoll, 1989; Lim & Cortina, 2005). Although the second discriminant function was not significant, it accounted for over 17% of the between-group differentiation and most likely helped to improve the classification results, albeit not significantly.

## 5. Discussion

This study examined the impact of direct sexual harassment and indirect bystander harassment on work attitudes, stress, withdrawal, and psychological health among part-time employees. Although much research has addressed the effects of sexual harassment, far less attention has been paid to how multiple forms of harassment jointly influence work outcomes, especially among part-time employees, whose work characteristics often differ from those of full-time employees. Bystander harassment experiences involve indirect harassment experiences in which the employee witnesses or knows about others being victimized (Schneider et al., 2000). The current study extends research in this area by examining how both direct sexual harassment and indirect bystander harassment experiences contribute to stress and strain within the workplace. Although both types of harassment were related to satisfaction, stress, and withdrawal, direct sexual harassment experiences had stronger effects across analyses.

Approximately 40% of participants reported experiencing at least one sexual or bystander harassment experience and were slightly upset or stressed by it. Among those who reported experiencing direct sexual harassment, most reported experiencing both gender harassment and unwanted sexual attention. Sexual coercion was reported much less frequently. This is consistent with previous research identifying sexual coercion as a less common but more severe form of harassment (Keplinger et al., 2019). Approximately 24% of participants reported experiencing both, while a much smaller proportion reported experiencing only one form of harassment. This supports the idea that organizational climates conducive to one form of harassment are also conducive to other forms (Ollo-López & Núñez, 2018). These results further suggest that harassment may be embedded within a deeper workplace culture, affecting not only those directly targeted but also those who observe or know about it happening around them.

Consistent with previous studies on the cumulative effects of harassment (Chan et al., 2008; Cortina & Areguin, 2021; Diez-Canseco et al., 2022; Draucker, 2019; Merkin & Shah, 2014; Sims et al., 2005), it was expected that sexual harassment and bystander harassment experiences would each be related to increased stress and withdrawal behaviors and decreased satisfaction and psychological health. The results partially support these hypotheses. Both sexual and bystander experiences were related to decreased supervisor satisfaction and increased work-related stress, work withdrawal, and job withdrawal. However, the predicted relationships between harassment experiences, psychological health, and coworker satisfaction failed to emerge. This may be a function of the sample’s part-time employment status. For example, part-time employees may not identify as closely with, be as emotionally attached to, or need to interact with coworkers as much as full-time employees (Conway & Briner, 2002; Sonnentag & Fritz, 2015; Thorsteinson, 2003). As such, it may be easier for part-time employees to disengage from fully participating in the day-to-day interactions required of full-time employees. In essence, their relationships may be more transactional and less emotional in nature. This may serve to decrease the psychological impact of harassment experiences; however, the environment may still be stressful for them. These factors should be examined more in depth in future research using more diverse populations.

In the set of moderation analyses, sexual and bystander experiences were predicted to have additive effects on work outcomes and psychological health, and these effects were expected to remain even after controlling for generalized negative dispositional tendencies. Sexual harassment experiences were significant predictors of decreased supervisor satisfaction, increased stress, and withdrawal. In contrast, bystander experiences did not independently predict work outcomes or psychological health status. Given part-time employees generally work fewer hours, their roles may involve only brief coworker interactions, which could buffer the impact of witnessing or hearing about harassment directed toward others in their workplace. They may not know their coworkers as well as full-time employees generally do, and as a result, they may experience less empathy toward their coworkers’ experiences and do not feel as personally threatened by such experiences (Miner & Eischeid, 2012; Reich & Hershcovis, 2015; Williams, 2007). Alternatively, the low base rate for both direct sexual harassment (raw score weighted index range: 0–3.16) and bystander harassment experiences (raw score weighted index range: 0–6.55) may have limited a strong test of interaction effects. Nevertheless, the absence of significant interaction effects aligns with models suggesting that additive effects are more typical than multiplicative effects in mistreatment research (Berdahl & Moore, 2006; Raver & Nishii, 2010). The obtained patterns warrant closer consideration, especially regarding the nonsignificant relations between harassment experiences and coworker satisfaction and psychological health.

Taken together, these findings indicate that sexual harassment is a more consistent predictor of part-time employees’ outcomes. This pattern suggests that direct experiences may draw more heavily on emotional and interpersonal resources than indirect exposure. Although both forms of harassment can signal a permissive organizational climate, the present results indicate that direct experiences more reliably undermine satisfaction, increase stress, and heighten withdrawal tendencies than indirect experiences. However, bystander exposure may still contribute to strain under conditions involving stronger or more frequent indirect mistreatment.

Discriminant function analysis was conducted to complement regression analyses. This supplemental analysis aids in determining whether work variables and psychological health can be used to reliably distinguish between harassment groups (no harassment, sexual harassment only, bystander harassment only, and both sexual and bystander harassment). One significant discriminant function accounting for over 80% of the between-group variance was identified. This model accurately classified 59% of the participants into one of the four harassment groupings. Supervisor satisfaction, stress, work withdrawal, and job withdrawal had the strongest loadings. This function maximally distinguished the no-harassment and bystander-only groups from the groups experiencing sexual harassment only and both sexual and bystander harassment. Moreover, the group that reported both forms of harassment was most strongly associated with this factor. This provides additional support that exposure to more than one form of harassment can contribute to satisfaction, stress, and withdrawal among part-time employees.

### Limitations, Implications, and Future Research

The limitations inherent in this study are similar to those across all cross-sectional studies, including the inability to infer the causal nature of relations. Thus, alternative models cannot be excluded. For example, employees who are more withdrawn from their organizations may be more likely to experience harassment, or perhaps the direction is bidirectional. Although the current research findings are consistent with those of prior research, experimental or longitudinal research designs are required to support such conclusions. Self-report data may increase the chances of self-report bias and method effects (Ollo-López & Núñez, 2018; Podsakoff et al., 2003). Additionally, the sample of part-time employed students may limit variability in employment contexts and harassment experiences. The sample was predominantly young, with a mean age of 18.79 years (*SD* = 1.44), and ethnically/racially homogeneous, with approximately 96% of the sample being White/Caucasian. This limitation affects the generalizability of the findings. Although these demographic characteristics are consistent with the local student population, they should be considered when discussing the broader applicability of these results to other populations. Greater diversity is needed to determine whether similar patterns emerge among older part-time employees, more experienced employees, and those who are more ethnically diverse.

Another potential limitation is the inclusion of different types of measurement scales. For example, for both direct sexual and bystander harassment experiences, indices were created in which the frequency of a given experience was multiplied by participants’ appraisal of how upset they were (direct sexual harassment) or how stressed they were (bystander harassment experiences) by each experience. For the outcome variables, a combination of frequency-based measures (work withdrawal and psychological health) and appraisal-based measures (e.g., satisfaction and stress) were included. However, it is not always clear how frequency counts map onto subjective evaluations of mistreatment, and this mismatch may introduce an additional level of complexity when assessing associations across various measurement approaches. Additionally, research in this area should examine whether the relationship between bystander harassment and outcomes varies across different types of part-time work. For example, some work roles require closer contact among employees and a greater focus on workgroup output. Both situations would require part-time employees to interact more with other employees and possibly increase the psychological closeness one feels toward other organizational members.

Despite these limitations, the findings of this study have valuable implications for organizations that employ part-time employees. These results suggest the importance of creating comprehensive anti-harassment policies that highlight and enforce zero tolerance for such behaviors. Organizations should consider implementing bystander intervention training to empower witnesses to take action and support those affected by harassment. Encouraging reporting through the implementation of anonymous reporting mechanisms for vulnerable employees, such as part-time employees, is important. Such an approach could also increase employees’ feelings of safety and support when reporting harassment or other negative workplace behaviors. Additionally, incorporating inclusive activities can increase employees’ attachment to the organization and decrease the likelihood of counterproductive work behaviors, withdrawal, and turnover, and increase positive interactions among employees and team members (Kessler et al., 2021).

## 6. Conclusions

This study examined the relations between sexual and bystander harassment experiences, work attitudes, stress, withdrawal, and psychological health among part-time employees. This population is often neglected in sexual harassment research, although it may be particularly vulnerable to such experiences. While acknowledging the limitations noted above, the current study adds to the limited research in this area and allows for a more nuanced understanding of the role of direct sexual harassment experiences and indirect bystander harassment experiences among part-time employees. Such research can also be useful for developing generalizable models of harassment.

## Figures and Tables

**Table 1:** Frequencies and percentages of participants reporting different types and combinations of sexual harassment (*n* = 309).

Harassment Experience	N	%
No Harassment	187	60.5%
Gender Harassment (GH) ONLY	31	10.0%
Unwanted Sexual Attention (USA) ONLY	20	6.5%
Sexual Coercion (SC) ONLY	1	0.3%
GH + USA	60	19.4%
GH + SC	1	0.3%
USA + SC	1	0.3%
GH + USA + SC	8	2.6%

Note. SEQ subscale categories: GH = Gender Harassment; USA = Unwanted Sexual Attention; SC = Sexual Coercion.

**Table 2:** Frequency and percentage of participants reporting sexual and bystander experiences (*n* = 309).

Experience Type	N	%
No sexual or bystander harassment experiences	172	55.7%
Sexual harassment ONLY	49	15.9%
Bystander harassment ONLY	14	4.5%
Both sexual and bystander harassment experiences	74	23.9%

**Table 3:** Means, standard deviations, zero-order correlations, and reliability estimates for study variables.

Variable	Mean	*SD*	1	2	3	4	5	6	7	8	9
1. SEQ	0.02	1.00	--								
2. Bystander	0.01	1.00	0.61 **	--							
3. SuperSat	0.46	0.40	0.28 **	0.18 **	(0.71)						
4. CowSat	0.34	0.37	0.10	0.02	0.31 **	(0.71)					
5. Job Stress	0.95	0.92	0.30 **	0.22 **	0.31 **	0.25 **	(0.85)				
6. Psy. Healt	4.00	0.77	−0.05	0.06	−0.09	−0.20 **	−0.07	(0.89)			
7. WorkWD	1.13	0.25	0.24 **	0.19 **	0.18 **	0.22 **	0.15 **	−0.09	(0.78)		
8. JobWD	2.57	0.95	0.22 **	0.14 **	0.34 **	0.29 **	0.30 **	−0.16 **	0.44 **	(0.73)	
9. Disp.	1.49	0.32	0.13 *	0.06	0.09	0.13 *	0.13 *	−0.23 **	0.05	0.10	(0.81)

Note. N = 304. ** *p* < 0.01, * *p* < 0.05. SEQ = Yeo-Johnson transformed sexual harassment experiences, Bystander = Yeo-Johnson transformed bystander harassment experiences, SuperSat = Reflect and log-transformed JDI supervisor satisfaction, CowSat = Reflect and log-transformed JDI coworker satisfaction, Psy. Healt = Psychological health, WorkWD = Log-transformed work withdrawal. JobWD = Job withdrawal; Disp. = Affective disposition. Reliability estimates are shown in parentheses along the diagonal.

**Table 4:** Summary of canonical discriminant functions.

Function	Eigenvalue	% of Variance	Canonical Correlation	Wilk’s Λ	χ^2^	df	*p*
1	0.173	80.2	0.384	0.817	61.14	18	<0.001
2	0.038	17.8	0.192	0.959	12.71	10	0.240
3	0.004	2.0	0.065	0.996	1.27	4	0.866

**Table 5:** Standardized canonical discriminant function coefficients.

Predictor Variable	Function 1	Function 2	Function 3
Job Stress	0.592	0.223	0.173
Work Withdrawal	0.486	0.367	0.054
Supervisor Satisfaction	0.392	−0.091	−0.536
Job Withdrawal	0.186	−0.337	0.274
Psychological Well-being	−0.103	0.832	0.324
Coworker Satisfaction	−0.408	−0.208	0.886

**Table 6:** Structure matrix for discriminant function loadings.

Predictor Variable	Function 1	Function 2	Function 3
Job Stress	0.683 *	0.049	0.320
Work Withdrawal	0.588 *	0.120	0.274
Supervisor Satisfaction	0.550 *	−0.202	−0.155
Job Withdrawal	0.538 *	−0.338	0.389
Psychological Well-being	−0.151	0.887 *	0.132
Coworker Satisfaction	0.052	−0.358	0.788 *

Note. Pooled within-groups correlations between discriminating variables and standardized canonical discriminant functions. * *p* < 0.05.

**Table 7:** Group centroids and classification results for harassment groups.

Group	*n*	Centroid (Function 1)	Centroid (Function 2)	Centroid (Function 3)	% Correctly Classified
No Harassment	172	−0.325	0.022	−0.026	89.5%
Sexual Harassment ONLY	49	0.363	−0.414	0.011	8.2%
Bystander Harassment ONLY	14	−0.402	0.081	0.288	0.0%
Both Sexual and Bystander Harassment	74	0.592	0.207	−0.001	31.1%
Overall Accuracy					58.6%
Cross Validated Accuracy					57.9%

Note. Centroids represent group means for each standardized canonical discriminant function. Classification accuracy (58.6%; cross-validated = 57.9%) exceeded both the proportional-by-chance rate (39.4%) and the 1.25 × chance criterion (49.3%). Press’s *Q*(3) = 17.6, *p* < 0.01, indicating that classification accuracy was significantly greater than chance.

## Data Availability

The original data presented in the study are openly available in Dataverse at https://doi.org/10.7910/DVN/NLZPJ9.
